# Effect of Cluster-Zone Leaf Removal at Different Stages on Cabernet Sauvignon and Marselan (*Vitis vinifera* L.) Grape Phenolic and Volatile Profiles

**DOI:** 10.3390/plants13111543

**Published:** 2024-06-02

**Authors:** Xuechen Yao, Yangpeng Wu, Yibin Lan, Yanzhi Cui, Tonghua Shi, Changqing Duan, Qiuhong Pan

**Affiliations:** 1 Center for Viticulture and Enology, College of Food Science and Nutritional Engineering, China Agricultural University, Beijing 100083, China; yaoxch@cau.edu.cn (X.Y.); 18810589600@163.com (Y.W.); lanyibin@cau.edu.cn (Y.L.); chqduan@cau.edu.cn (C.D.); 2 Key Laboratory of Viticulture and Enology, Ministry of Agricultural and Rural Affairs, Beijing 100083, China; 3 Bodega Langes Co., Ltd., Qinghuangdao 066600, China; cuiyz999@sina.com (Y.C.); shitonghua0101@163.com (T.S.)

**Keywords:** cluster-zone leaf removal, Cabernet Sauvignon, Marselan, phenolic compounds, volatile compounds

## Abstract

This study investigated the effect of leaf removal at three stages of grape development on the phenolic and volatile profiles of Cabernet Sauvignon and Marselan grapevines for two consecutive years in the Jieshi Mountain region, an area of eastern China with high summer rainfall. The results indicated that cluster-zone leaf removal generally reduced the titratable acidity of both varieties, but did not affect the total soluble solids of grape berries. Leaf-removal treatments increased the anthocyanin and flavonol content of berries in both varieties. However, in Cabernet Sauvignon, leaf removal negatively affected the norisoprenoid compounds, with a more pronounced impact observed when the leaf removal was conducted at an early stage. This negative effect may be related to a decrease in the levels of violaxanthin and neoxanthin, potential precursors of vitisprine and *β*-damascenone. In contrast, the removal of leaves had no effect on the norisoprenoid aroma of Marselan grapes.

## 1. Introduction

Phenolic and volatile compounds are important secondary metabolites in wine grapes that are extracted during the maceration and fermentation processes, providing color and aroma to the wine. Generally, phenolic metabolites in wine grapes originate from the flavonoid metabolic pathway, while aroma metabolites are derived from three distinct metabolic pathways. These pathways include the lipoxygenase pathway, which produces C6/C9 compounds and volatile thiol precursors; the plastidic methylerythritol phosphate and cytosolic mevalonic acid pathways, which generate monoterpenes and C13-norisoprenoids; and the phenylalanine pathway, which produces aromatic compounds such as benzenoids and phenylpropanoids [1]. These secondary metabolic pathways are influenced by a range of biotic and abiotic factors, including sunlight, temperature, precipitation, microbial diseases, and insect damage [2,3,4]. In order to enhance grape growth conditions, grape growers around the world implement numerous agronomic techniques, including leaf removal, canopy training systems, foliar fertilization, deficit irrigation, and inter-row planting [5,6,7]. These practices ultimately alter the secondary metabolism of grape berries, leading to an improvement in the quality of the colour and aroma.

Cluster-zone leaf removal, also known as basal leaf removal, is a viticulture practice aimed at improving the microclimate of grapevines. This practice aims to enhance air circulation, increase cluster exposure, reduce pests and diseases, and improve berry quality. Previous studies have indicated that the effects of cluster-zone leaf removal on grape-berry ripening and phenolic content are inconsistent, possibly due to varietal and treatment-period differences. Investigations of Sauvignon Blanc grapes demonstrated that removing leaves around veraison (two weeks before, during, and two weeks after) may lead to a decrease in levels of both titratable acid (TA) and total soluble solids (TSS) in grape berries [8]. Conversely, removing leaves at an earlier phenological stage (15 days before and after flowering) did not significantly affect either TA or TSS levels in ripe grape berries [9,10]. Cluster-zone leaf removal of Pinot Noir at different stages, including flowering, pea grain size, and cluster closure, has been shown to promote the accumulation of anthocyanins and flavonols. Furthermore, early leaf removal has been found to result in higher anthocyanin levels [11]. Similar results have been observed in leaf-removal treatments of Cabernet Sauvignon grapes performed at the beginning and end of veraison [5,12]. However, for Merlot grapes, leaf removal before and after flowering did not result in any discernible changes in the levels of anthocyanins and tannins [13].

Several studies have demonstrated the diverse effects of cluster-zone leaf removal on grape volatile compounds, with the effects varying depending on the grape variety, climate, treatment stage, and intensity. Most of these studies have indicated that the removal of leaves has the effect of promoting the synthesis of monoterpenes, specifically *α*-terpineol and linalool, and their oxidised derivatives [10,11,14]. One study observed an increase in the total amount of monoterpenes in the group with 50% leaf removal during veraison, while no significant difference was observed in the group with 100% defoliation [8]. Furthermore, leaf removal during the pepper-corn-size stage and veraison resulted in a reduction in the total terpene content in an intensely light, dry–hot climate [15], indicating that overexposure could have a negative impact on terpene accumulation. A number of studies have indicated that leaf removal from the cluster zone can lead to increased levels of norisoprenoids [11,14,16]. This is thought to be linked to increased synthesis of carotenoids [10]. Nevertheless, two studies on Cabernet Sauvignon grapes demonstrate that the *β*-damascenone levels were markedly lower in the defoliated group than in the control group [15]. In conclusion, the multifaceted impact of leaf removal on grape aroma necessitates the selection of an appropriate processing timing and intensity based on the intended purpose and the grape-production region.

We examined the effects of cluster-zone leaf-removal treatments on Cabernet Sauvignon grapes at three developmental stages in 2021. The following year, our study was expanded to include not only Cabernet Sauvignon grapes but also Marselan grapes. The objective of this study was to evaluate the impact of these treatments on the phenolic and volatile profiles of both varieties in a wine-producing region with a typical continental monsoon climate and annual rainfall mainly occurring from July to September. The objective was to devise a strategy to enhance the quality of the colouration and aroma of the grapes.

## 2. Results and Discussion

### 2.1. Meteorological Conditions and Grape Physicochemical Parameters

**Figure 1:**
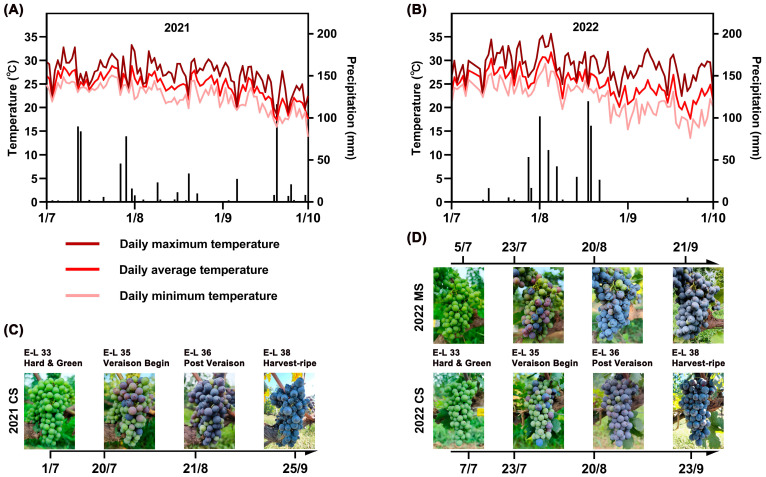
Daily maximum, average, and minimum temperatures (lines) and precipitation (histograms) recorded from 1 July to 1 October at the Chateau Langes weather station in 2021 (**A**) and 2022 (**B**). Time axis of Cabernet Sauvignon and Marselan berry development and ripening in 2021 (**C**) and 2022 (**D**).

Meteorological data were collected from two berry-growing seasons in Jieshishan region (Figure 1 and Table S1). As the Cabernet Sauvignon experiment spanned two years, while the Marselan experiment spanned only one year, this comparative analysis will concentrate on the differences in meteorological conditions and their effects during the growing season of Cabernet Sauvignon grapes between the two years. The climate was observed to be warmer in 2022 relative to 2021, with the average daily temperatures registering at 0.5 °C higher between 1 July and 1 October. Specifically, during the periods from the hard green berry stage to the beginning of veraison and from post-veraison to harvest, the average daily temperatures increased by 1.5 °C and 1.4 °C, respectively (Table S1). In 2022, during the veraison stage, there were two days when the temperature peaked above 35 °C, whereas no such instances were recorded in 2021. The higher temperatures resulted in an accelerated grape-ripening process, which led to an increase in TSS and a decrease in TA in Cabernet Sauvignon grapes in 2022 (Table 1). In 2022, the quantity of precipitation exceeded that in 2021 by 32.1 mm. Additionally, the distribution of rainfall during the berry-development phase exhibited variability between the two years. In 2021, precipitation was primarily observed during the green-berry and ripening stages, while in 2022, it was concentrated during the veraison stage (Figure 1).

Table 1 presents the physicochemical parameters of grape berries at harvest following cluster-zone leaf removal at various stages. The ANOVA analysis revealed that the leaf-removal treatment exerted a profound effect on the levels of titratable acids (TA) in the berries. In Cabernet Sauvignon grapes, the LR2 treatment resulted in significantly lower levels of titratable acid (TA) compared to the control in 2021. Furthermore, all three periods of leaf-removal treatments significantly diminished TA levels in 2022, with LR2 resulting in the lowest average levels. Moreover, the pH value was found to be significantly lower in the control compared to LR2 and LR3 in 2021, whereas no significant disparity was observed between the treatments and the control in 2022. Despite annual variations, these findings indicate that cluster-zone leaf-removal treatments typically decrease acidity in Cabernet Sauvignon berries and suggest that leaf removal during veraison could be more beneficial. A comparable pattern was observed in the 2022 Marselan berries of Cabernet Sauvignon grapes from the same year, with LR1 and LR2 possessing significantly lower titratable acidity than the control and no significant pH variation between the groups. The observed decrease in berry TA is consistent with the expected effects of leaf removal on bolstering malic acid degradation, as excessive sunlight exposure results in elevated temperatures within the cluster zone [7,17]. Furthermore, the removal of leaves from the cluster zone did not result in a significant impact on the 100-berry weight or soluble solids content of the grapes.

### 2.2. Impact of Cluster-Zone Leaf Removal on Grape Phenolic Profiles

#### 2.2.1. Anthocyanins

Five monomeric anthocyanins and their acetylated and coumarylated forms were identified (Table 2). The total concentration of anthocyanins in Marselan grapes was approximately 30% higher than in Cabernet Sauvignon grapes, and Malvidin-type anthocyanins were the most abundant in both cultivars. Additionally, Marselan grapes exhibited higher proportions of methylated and coumarylated anthocyanins and a lower proportion of acetylated forms. In 2021, all three LR treatments increased the concentration of malvidin-3-*O*-glucoside and its acylated and coumarylated forms. However, no statistically significant differences were observed in the total anthocyanin concentration between the control and the three LR groups. In the Cabernet Sauvignon grapes of 2022 vintage, both LR1 and LR2 significantly increased the total anthocyanin concentration compared to LR3 and the control group. LR2 exhibited the most pronounced effect, with significantly higher concentrations of all anthocyanins compared to the control group. A comparable pattern was observed in the Marselan grapes of 2022 vintage, with all three LR treatments resulting in an approximate 10% increase in the total anthocyanin concentration. Among the treatments, LR2 resulted in the highest concentration of malvidin-type anthocyanins, while LR1 resulted in the highest concentration of other types of anthocyanins. A number of studies have indicated that leaf removal promotes anthocyanin accumulation by enhancing light exposure and temperature [5,11,12], and our investigation also verifies these conclusions. Furthermore, it has been proposed that elevated temperatures could enhance the acylation and methylation rates of anthocyanins [17]. However, no such phenomenon was observed between the two years, nor were any consistent patterns identified among the different leaf-removal treatments.

#### 2.2.2. Flavonols

A total of 15 flavonols were identified in the grape skins (Table 3), with notable variations in concentration observed between the two years. The total concentration of flavonols in 2022 Cabernet Sauvignon grapes was approximately 45% higher than that in 2021 grapes, which could be attributed to the differences in irradiance between the two years [18]. Furthermore, the proportions of flavonols differed between the two varieties, with Cabernet Sauvignon grapes having a higher proportion of kaempferol- and quercetin-type flavonols, while Marselan grapes exhibited higher proportions of myricetin, laricitrin, and syringetin-type flavonols. These findings indicate that Marselan grapes tend to accumulate F3′5′H flavonols more than Cabernet Sauvignon grapes.

Previous studies have indicated that flavonols are reliable indicators for assessing canopy structure and solar radiation [19]. Consistent with these findings, our study showed that the concentrations of most flavonol compounds, except for quercetin-3-*O*-rutinoside, increased in the three LR treatments of Cabernet Sauvignon grapes in 2021. Furthermore, the flavonol concentrations in the three LR treatments of Cabernet Sauvignon grapes in 2022 were found to be significantly higher than those in the control group. It has been demonstrated that flavonols can contribute to the copigmentation of anthocyanins during winemaking [20], which suggests that LR treatments may potentially enhance the quality and stability of the colour of Cabernet Sauvignon wines. In 2022 Marselan grapes, the total flavonol concentration was significantly higher in the LR1 treatment compared to the control group, while the LR2 and LR3 treatments exhibited lower flavonol concentrations than the control group. Furthermore, the composition of flavonols differed between the LR treatments and the control group. The LR treatments resulted in higher proportions of kaempferol and quercetin, whereas the control group exhibited higher proportions of myricetin, laricitrin, and syringetin-type flavonols.

#### 2.2.3. Flavan-3-Ols

A total of six free flavan-3-ols were identified and the composition of proanthocyanidins was investigated. The results showed that LR treatment had no impact on the total concentration of free flavan-3-ols, which is consistent with previous studies on the Cabernet Sauvignon [18], Pinot Noir [11] and Sangiovese [21] grapes. Moreover, no consistent trend in the impact of LR treatment on total flavan-3-ols across two distinct varieties over two years (Table S2). In 2021, the total proanthocyanidins and flavan-3-ols in Cabernet Sauvignon grapes treated with LR ware found to be higher than in the control group, with LR2 exhibiting the highest content in both extension units and terminal units. In 2022, a significant difference was observed in the concentrations of total proanthocyanidins and flavon-3-ols between LR2 and LR3 in Cabernet Sauvignon grapes. However, the differences between the LR treatment groups and the control group were not statistically significant. Furthermore, no significant differences in total proanthocyanidins and flavon-3-ols were observed between the different groups of Marselan grapes in 2022. In a previous study, the impact of consecutive three-year LR treatments on the proanthocyanidins in Cabernet Sauvignon grapes did not exhibit a consistent trend [18]. This indicates that the impact of LR treatments on the composition of proanthocyanidins may be relatively insignificant and is likely to be influenced by other factors.

### 2.3. Grape Volatile Aromas of Grapes and Their Glycosylated Precursors

#### 2.3.1. C6/C9 Compounds

The green-leaf odours present in wine are primarily derived from the C6/C9 compounds extracted from grapes [22,23]. A total of 14 C6/C9 compounds were detected, including 10 free-form volatiles and 4 glycoside-bound volatiles, certain of which exhibited significant differences between the two varieties (Table 4). Marselan berries were found to contain almost twice the concentrations of bound 1-hexanol, (*Z*)-3-hexen-1-ol, and 1-nonanol compared to the Cabernet Sauvignon grapes. Conversely, the Cabernet Sauvignon variety exhibited higher levels of free C6/C9 alcohols, specifically (*E*)-2-hexen-1-ol, (*E*)-3-hexen-1-ol, and 1-hexanol.

The LR treatment resulted in elevated levels of free C6 alcohols in Cabernet Sauvignon grapes, with LR2 in particular exhibiting the most pronounced impact on (*Z*)-3-hexen-1-ol and (*E*)-2-hexen-1-ol levels in both years. LR1 and LR3 also increased levels of 1-hexanol and (*E*)-2-hexen-1-ol in 2021, although to a lesser extent than did LR2. Conversely, LR1 and LR2 reduced levels of C6/C9 aldehydes, including hexanal, (*E*)-2-hexenal, nonanal and (*E*,*E*)-2,4-hexadienal in 2021. This suggests that LR treatments facilitate the metabolic conversion of C6 aldehydes to C6 alcohols, potentially through the upregulation of *VvADH* expression [2,24]. This finding is comparable to those of our prior investigations on Cabernet Sauvignon grapes conducted in the Xinjiang region of northwestern China, where leaf removal during veraison resulted in elevated levels of various C6 alcohols, including (*Z*)-3-hexen-1-ol, (*E*)-3-hexen-1-ol, (*E*)-2-hexen-1-ol, and 1-hexanol. The increased expression of the alcohol dehydrogenase gene (*VvADH1*) was identified as the underlying cause [20]. In contrast, LR treatments resulted in significantly higher levels of C6/C9 aldehydes in Marselan berries compared to Cabernet Sauvignon grapes. All three leaf-removal treatments led to increased concentrations of (*E*)-2-hexenal, with LR1 and LR3 resulting in approximately 50% higher hexanal levels compared to the control.

#### 2.3.2. Terpenes

The presence of terpenes in grapes is responsible for imparting floral aromas to wines [22]. Nine terpenes were identified in the study. The results showed that the total terpene content was higher in Marselan grapes, with higher levels of *p*-cymene in its free form, α-terpineol and *β*-citronellol in the bound form. In contrast, in Cabernet Sauvignon grapes, only levomenthol was present at higher levels (Table 5).

**Table 4:** Composition of C6/C9 compounds (μg/kg) in Cabernet Sauvignon and Marselan grapes at different stages of leaf-removal treatment from 2021 to 2022.

Compounds	Vintage and Variety	LR1	LR2	LR3	CK
1-Hexanol	2021 Cabernet Sauvignon	874.97 ± 251.56 ab	1085.27 ± 73.2 a	676.77 ± 134.92 b	341.77 ± 129.16 c
	2022 Cabernet Sauvignon	220.17 ± 26.08	225.96 ± 20.03	207.19 ± 44.75	253.82 ± 19.91
	2022 Marselan	83.72 ± 6.07	117.07 ± 8.24	96.58 ± 42.14	84.09 ± 21.44
(*E*)-3-Hexen-1-ol	2021 Cabernet Sauvignon	1.76 ± 0.61 b	2.71 ± 0.35 a	1.12 ± 0.28 bc	0.63 ± 0.27 c
	2022 Cabernet Sauvignon	0.58 ± 0.1	0.59 ± 0.17	0.55 ± 0.13	0.51 ± 0.11
	2022 Marselan	0.25 ± 0.06	0.28 ± 0.02	0.27 ± 0.01	0.26 ± 0.05
(*Z*)-3-Hexen-1-ol	2021 Cabernet Sauvignon	20.38 ± 5.37 ab	25.46 ± 2.93 a	16.06 ± 0.69 b	22.07 ± 5.77 ab
	2022 Cabernet Sauvignon	20.96 ± 1.88 b	32.48 ± 3.28 a	23.93 ± 1.95 b	18.96 ± 5.76 b
	2022 Marselan	3.34 ± 1.19 b	6.9 ± 0.95 a	2.75 ± 0.46 b	2.07 ± 0.53 b
(*E*)-2-Hexen-1-ol	2021 Cabernet Sauvignon	34.03 ± 9.32 b	48.4 ± 5.91 a	28.54 ± 3.75 b	12.54 ± 2.92 c
	2022 Cabernet Sauvignon	19.18 ± 3.51 b	23.42 ± 2.38 a	19.87 ± 2.68 b	18.92 ± 1.35 b
	2022 Marselan	3.96 ± 0.51	5.81 ± 0.91	4.28 ± 1.29	3.99 ± 0.92
1-Nonanol	2021 Cabernet Sauvignon	0.3 ± 0.32	0.16 ± 0.07	0.13 ± 0.03	0.04 ± 0.01
	2022 Cabernet Sauvignon	0.01 ± 0.01 b	0.01 ± 0.01 b	0.01 ± 0.01 b	0.02 ± 0.01 a
	2022 Marselan	0.01 ± 0.01	0.01 ± 0.01	0.01 ± 0.01	0.01 ± 0.01
Hexanal	2021 Cabernet Sauvignon	904.47 ± 137.91 c	807.81 ± 77.27 c	1168.99 ± 120.22 b	1599.76 ± 106.62 a
	2022 Cabernet Sauvignon	1741.02 ± 358.73	1715.35 ± 292.47	1689.5 ± 368.8	1601.67 ± 136.81
	2022 Marselan	1369.63 ± 246.2 a	968.46 ± 191.35 bc	1266.12 ± 78.37 ab	880.32 ± 136.6 c
(*E*)-2-Hexenal	2021 Cabernet Sauvignon	841.82 ± 26.55 c	1046.99 ± 82.52 b	1218.98 ± 144.06 b	1468.17 ± 103.45 a
	2022 Cabernet Sauvignon	2370.67 ± 207.95	2732.25 ± 594.85	2281.23 ± 393.2	2654.72 ± 379.37
	2022 Marselan	2047.78 ± 207.24 a	1935.81 ± 136.68 a	1984.76 ± 186.49 a	1556.95 ± 116.68 b
Nonanal	2021 Cabernet Sauvignon	1.17 ± 0.16 b	0.96 ± 0.28 b	2.16 ± 0.32 a	1.9 ± 0.55 a
	2022 Cabernet Sauvignon	1.21 ± 0.22	1.8 ± 0.33	1.14 ± 0.19	1.47 ± 0.5
	2022 Marselan	1 ± 0.2 ab	0.69 ± 0.09 ab	1.01 ± 0.14 a	0.67 ± 0.22 b
(*E*,*E*)-2,4-Hexadienal	2021 Cabernet Sauvignon	3.24 ± 1.56 b	4.16 ± 1.54 b	5.48 ± 0.57 ab	7.02 ± 0.62 a
	2022 Cabernet Sauvignon	12.11 ± 2.14	12.21 ± 2.05	10.44 ± 1.78	11.58 ± 1.29
	2022 Marselan	10.08 ± 1.34 a	9.28 ± 0.9 ab	10.2 ± 0.41 a	7.79 ± 0.18 b
Hexanoic acid	2021 Cabernet Sauvignon	3.04 ± 0.38	2.8 ± 0.42	2.94 ± 0.18	2.7 ± 0.45
	2022 Cabernet Sauvignon	1.62 ± 1.54 b	4.6 ± 2.31 ab	4.81 ± 0.69 ab	5.33 ± 2.12 a
	2022 Marselan	2.97 ± 0.4 b	5.03 ± 1.3 a	3.12 ± 0.19 b	3.9 ± 0.72 ab
1-Hexanol (Bound)	2021 Cabernet Sauvignon	15.19 ± 2.64	11.36 ± 1	13.81 ± 3.6	10.85 ± 3.68
	2022 Cabernet Sauvignon	21.79 ± 1.65	20.39 ± 4.45	23.55 ± 4.77	24.28 ± 3.79
	2022 Marselan	47.37 ± 9.74	51.83 ± 5.79	43.23 ± 2.26	52.98 ± 8.87
(*Z*)-3-Hexen-1-ol (Bound)	2021 Cabernet Sauvignon	3.86 ± 0.52 a	2.21 ± 1.24 b	2.61 ± 0.63 ab	2.52 ± 0.72 ab
	2022 Cabernet Sauvignon	8.82 ± 1.05	7.26 ± 1.19	9.74 ± 1.73	7.92 ± 1.3
	2022 Marselan	30.93 ± 2.91	33.23 ± 6.82	30.03 ± 9.57	34.51 ± 3.56
(*E*)-2-Hexen-1-ol (Bound)	2021 Cabernet Sauvignon	10.78 ± 3.01	8.07 ± 1.22	7.94 ± 0.72	9.46 ± 2.82
	2022 Cabernet Sauvignon	9.69 ± 6.1 ab	5.22 ± 0.35 b	7.06 ± 0.97 ab	13.56 ± 3.74 a
	2022 Marselan	5.92 ± 0.12	6.36 ± 1.01	6.61 ± 2.62	6.62 ± 0.73
1-Nonanol (Bound)	2021 Cabernet Sauvignon	0.05 ± 0.01 b	0.05 ± 0.01 b	0.07 ± 0.01 a	0.05 ± 0.01 b
	2022 Cabernet Sauvignon	0.07 ± 0.01 a	0.03 ± 0.01 c	0.05 ± 0.01 b	0.06 ± 0.01 ab
	2022 Marselan	0.1 ± 0.02 b	0.15 ± 0.03 a	0.1 ± 0.01 b	0.13 ± 0.04 ab
Total C6/C9 Alcohol (Free)	2021 Cabernet Sauvignon	931.27 ± 217.85 a	1162 ± 62.43 a	722.61 ± 111.78 ab	377.05 ± 108.29 b
	2022 Cabernet Sauvignon	260.9 ± 25.03	282.46 ± 20.62	251.55 ± 38.1	292.23 ± 13.08
	2022 Marselan	122.62 ± 45.8	130.07 ± 6.52	103.89 ± 35.79	105.43 ± 26.47
Total C6/C9 Aldehyde	2021 Cabernet Sauvignon	1750.7 ± 125.44 c	1859.92 ± 81.62 c	2395.61 ± 200.13 b	3076.84 ± 169.43 a
	2022 Cabernet Sauvignon	4125.02 ± 464.15	4461.61 ± 712.14	3982.32 ± 623.14	4269.42 ± 413.69
	2022 Marselan	3428.49 ± 368.16 a	2914.2 ± 260.77 ab	3262.09 ± 207.93 a	2445.73 ± 70.64 b
Total C6/C9 Alcohol (Bound)	2021 Cabernet Sauvignon	29.88 ± 3.25	21.69 ± 1.66	24.43 ± 2.79	22.88 ± 5.72
	2022 Cabernet Sauvignon	40.36 ± 5.15	32.9 ± 4.32	40.4 ± 5.01	45.83 ± 2.55
	2022 Marselan	86.97 ± 7.88	91.57 ± 5.85	79.97 ± 11.71	94.24 ± 10.49
Total C6/C9 Compounds	2021 Cabernet Sauvignon	2714.9 ± 180.58 b	3046.4 ± 78.31 ab	3145.6 ± 148.2 ab	3479.48 ± 266.47 a
	2022 Cabernet Sauvignon	4427.91 ± 440.03	4781.57 ± 710.35	4279.08 ± 588.76	4612.81 ± 408.39
	2022 Marselan	3641.05 ± 346.82 a	3140.8 ± 265.17 ab	3449.08 ± 222.87 a	2650.3 ± 35.54 b

Note: Mean ± SD are presented (*n* = 3); Lowercase letters indicate significant differences at *p* < 0.05 according to Tukey’s test.

Sunlight upregulates the expression of crucial genes (*VvDXS* and *VvTPS*) and transcription factors (*VvMYB24*) involved in the biosynthesis of grape terpenes, resulting in increased terpene concentrations [2,24,25]. A numbers of studies have indicated that LR treatment has a greater effect on glycosylated terpenes than free terpenes [11,15,26]. In this study, it was observed that the total concentration of glycosylated terpenes in Cabernet Sauvignon grapes significantly increased with LR1 treatment in both years, while the levels of free terpenes such as linalol, levomenthol, and *α*-terpineol decreased. Furthermore, in 2022, Cabernet Sauvignon grapes exhibited decreased free terpene levels and displayed heightened responsiveness to leaf-removal treatments compared to 2021, which can be attributed to higher temperatures throughout the 2022 berry growth season. Previous research indicates that elevated temperatures can impede the accumulation of free terpenes [27]. Nevertheless, the impact of LR treatment on the terpene levels of Marselan berries was minimal.

#### 2.3.3. Norisoprenoids and Carotenoids

The norisoprenoids present in grapes contribute to the floral and fruity aromas of wines [23]. In this study, seven norisoprenoids were identified (Text 6). The study revealed that Marselan grapes exhibited a larger variety and total content of norisoprenoids, with the maximum content of *β*-damascenone nearly twice as high as that in Cabernet Sauvignon grapes. This finding is consistent with the previous study where Marselan wines had strong floral and fruity aromas [28,29]. Carotenoids, which serve as direct precursors for these aromas in grape berries, are converted to norisoprenoids by carotenoid cleavage dioxygenases (CCDs) [1]. A total of six carotenoids were identified, with Marselan grapes exhibiting elevated levels of *β*-carotene, xanthophyll, violaxanthin, and neoxanthin in comparison to Cabernet Sauvignon grapes (Text 6). The total carotenoid content in Marselan grapes was approximately 1.5 times higher than that in Cabernet Sauvignon grapes from the same vintage. The elevated concentration of carotenoids in Marselan grapes may be a contributing factor to its superior norisoprenoid content.

The LR treatment had a significant impact on the norisoprenoid concentrations in Cabernet Sauvignon berries, resulting in a decreased levels of the majority of norisoprenoids. Notably, *β*-damascenone was particularly affected, constituting only 12–15% of the control in LR1 for both years. Furthermore, the concentration of *β*-damascenone exhibited a declining trend with earlier leaf removal. It can be postulated that an extended exposure of the berry’s surface may be unfavorable for the accumulation of norisoprenoids, in agreement with the results pf prior research [15]. Violaxanthin and neoxanthin, respectively, are speculated to be precursors of vitispirane and *β*-damascenone [30,31]. Violaxanthin is a crucial component of the xanthophyll cycle and plays a significant role in plant photoprotection. In response to excessive light, violaxanthin is transformed into zeaxanthin via antheraxanthin by violaxanthin de-epoxidase (VDE). Conversely, under low-light conditions, zeaxanthin is converted into antheraxanthin and violaxanthin by the activity of zeaxanthin epoxidase (ZEP) [32]. In both years of our study on Cabernet Sauvignon grapes, lower levels of violaxanthin were observed in the LR treatment, and the proportion of violaxanthin within the xanthophyll cycle was also lower in the LR1 and LR2 treatments. However, there were no significant differences in comparison to the control (Table 6). A similar trend was observed for neoxanthin, which is a downstream metabolite of violaxanthin. Therefore, we hypothesize that the reduction in vitispirane and *β*-damascenone following LR treatments may be associated with the decrease in their precursors, violaxanthin and antheraxanthin. This pattern was not observed in Marselan berries, in which LR1 solely reduced the level of glycosylated *β*-damascenone. Furthermore, the LR treatment had no effect on either total norisoprenoids or carotenoids, in contrast to the results observed in Cabernet Sauvignon grapes during the same year (Text 6).

#### 2.3.4. Aromatic Compounds

The volatile aromatic compounds resulting from the metabolism of phenylalanine contribute to the rose-and-honey aroma that is crucial for both winemaking and table grapes. A total of five free and three bound aromatic compounds were identified (Supplementary Table S3). The results showed that the LR treatment had a more pronounced impact on the levels of aromatic alcohols in relation to the vintage, whereas its effect on aromatic aldehydes was more dependent on grape variety. In 2021, the LR1 treatment resulted in an increased accumulation of aromatic alcohols. However, during the 2022 harvest, both Cabernet Sauvignon and Marselan grapes exhibited reduced levels of aromatic alcohols in the LR1 treatment group in comparison to the control. The results demonstrated that LR treatment had no significant impact on the levels of aromatic aldehydes in Marselan grapes. However, it generally reduced the levels of aromatic aldehydes in Cabernet Sauvignon berries. Additionally, the LR treatment resulted in a reduction in the levels of glycosylated phenylethyl alcohol and benzyl alcohol in Cabernet Sauvignon grapes from the 2022 vintage.

## 3. Materials and Methods

### 3.1. Reagents and Standards

Analytical-grade chemicals including sodium chloride, glucose, citric acid, sodium hydroxide, disodium hydrogen phosphate, potassium hydrogen phthalate, and anhydrous copper sulphate were purchased from Beijing Chemical Factory in Beijing, China. Chromatography-grade solvents, for example, methanol (≥99.9%) and dichloromethane (≥99.9%), were procured from Honeywell, located in Marris Township, NJ, USA, while formic acid (≥99%) was procured from ROE Scientific in Newark, NJ, USA. Standards of volatile compounds were purchased from commercial vendors, including Sigma-Aldrich (St. Louis, MO, USA), TCI Shanghai (Shanghai, China), Extrasynthèse Chemical S.A.S. (Genay, France), and Shanghai Standard Technology Co., Ltd. (Shanghai, China).

### 3.2. Vineyard Experimental Design

In 2021 and 2022, a set of experiments that involved leaf removal were conducted in the commercial vineyards of Chateau Langes, which are located at 119°25′ N, 39°76′ E, within Jieshishan county. These vineyards have a 0.3% incline gradient in the western direction and predominantly comprise sandy loam soil. The study examined *Vitis vinifera*, L. cv Cabernet Sauvignon and Marselan cultivars, both of which are self-rooted and grown using the pruning training system called modified vertical spur positioning (M-VSP). The grapevine rows were arranged in a nearly south–north orientation and spaced at 2 m × 1 m (vine × row). The investigation with Cabernet Sauvignon grapes was conducted over a two-year period (2021 and 2022), whereas the experimentation with Marselan grapes was limited to the 2022 growing season.

For the study, a group of 24 grapevines with uniform growth within a designated row were selected. The vines were divided into four experimental groups, each based on the timing of leaf-removal interventions. Six basal leaves were removed to completely expose the grapes. Leaf removal occurred during specific periods, including at the hard green berry stage (designated as LR1), at the beginning of veraison (LR2), 25 days after veraison (LR3). A control group underwent no leaf removal. Each row was a cluster of biological replicates, resulting in a total of three replicative rows. During the commercial harvest period, 300 individual berries were collected randomly from each biological replicate. Out of these, 50 berries were subjected to physicochemical analysis, while the rest were frozen in liquid nitrogen and stored at −80 °C until the analysis was completed.

### 3.3. Meteorological Data

Temperature, humidity, and precipitation data were collected by a weather station (MC-QXSQ, Nongchuang, Beijing, China) situated in the vineyard. Data were recorded at 30-min intervals. Growing degree–days (GDD10) were calculated by summing the daily average of maximum and minimum temperatures above 10 °C. Daily maximum temperatures of at least 35 °C were identified as extreme heat waves, and the researchers recorded the number of days with such high temperatures during the period of berry development.

### 3.4. Physicochemical Parameter Measurement

In each biological replicate, 50 berries were repeatedly weighed and manually squeezed to obtain the juice used for other physicochemical analyses. The total soluble solids (TSS) of the juices were measured using a pocket Brix refractometer (PAL-1, ATAGO, Tokyo, Japan). The pH was assessed using a calibrated pH meter (FE20, Mettler Toledo, Greifensee, Switzerland). Total titratable acid content was determined by titration with 0.1 mol/L NaOH, using phenolphthalein as an indicator. Titratable acidity was expressed in g/L tartaric acid.

### 3.5. Quantitative Analysis of Phenolic Compounds

#### 3.5.1. Extraction of Phenolic Compounds from Grape Skins

The extraction of anthcyanosides and flavonols from grape skins was conducted following the methods used in our previous report [33,34]. Frozen grape-skin powder (0.100 ± 0.002 g) was mixed with 1 mL of a 50% (*v*/*v*) methanol–water solution. Ultrasonic extraction was performed for 20 min and was followed by centrifugation at 8000 *g* for 10 min. The supernatant was collected, and the residue was extracted twice.

The extraction method for flavan-3-ols from grape skins was the same as in previous studies [35,36]. To extract free flavan-3-ols, frozen grape-skin powder (0.100 ± 0.002 g) was mixed with 1 mL of a 70% acetone–water solution containing 0.5% ascorbic acid (*w*/*v*). Ultrasonic extraction was performed for 20 min and was followed by centrifugation at 8000 *g* for 10 min. The residue was extracted twice, and the supernatant was evaporated to dryness using nitrogen gas. The residue was then dissolved in 200 μL of a methanol solution containing 1% HCl, and 200 μL of a sodium acetate aqueous solution was added for neutralization. The neutralized extract was used to determine the content of free flavan-3-ols.

To extract proanthocyanidins, frozen grape-skin powder (0.500 ± 0.001 g) was mixed with 0.5 mL of a phloroglucinol buffer solution containing 0.5% ascorbic acid (*w*/*v*). The mixture was heated at 50 °C in a water bath for 20 min, following which 0.5 mL of a sodium acetate solution was added to stop the reaction. Centrifugation was carried out at 8000 *g* for 10 min, and the supernatant was collected. The residue was extracted twice.

#### 3.5.2. Analysis of Phenolic Compounds

The analysis of phenolic compounds in grape skins was conducted using high-performance liquid chromatography coupled with triple quadrupole mass spectrometry (HPLC-QqQ-MS/MS, Agilent 1200 series HPLC system, Agilent 6410 QqQ-MS). HPLC separation was performed on a Poroshell 120 EC-C18 column (2.1 × 150 mm, 2.7 μm, Agilent Technologies, Santa Clara, CA, USA), with the mobile phase consisting of (A) water containing 0.1% formic acid (FA) and (B) acetonitrile/methanol (50:50, *v*/*v*) containing 0.1% FA. Electrospray ionization (ESI) was used as the ionization source, with positive ion mode for the analysis of anthcyanosides and flavonols and negative ion mode for flavan-3-ols. The specific chromatographic and mass spectrometric conditions have been described in previous articles [36,37].

#### 3.5.3. Qualitative and Quantitative Analysis of Phenolic Compounds

Compounds were identified by comparing the retention times and qualitative transition ions of standards in a self-built library. Anthcyanosides were quantified using Malvidin-3-*O*-glucoside as an external standard. Flavonols were quantified using isorhamnetin-3-*O*-glucoside, kaempferol-3-*O*-glucoside, kaempferol-3-*O*-galactoside, myricetin-3-*O*-galactoside, quercetin-3-*O*-glucoside, and syringetin-3-*O*-glucoside as external standards. Flavanols were quantified using (+)-catechin, (−)-epicatechin, (−)-epicatechin gallate, (−)-epigallocatechin gallate, (−)-epigallocatechin, and (+)-gallocatechin as external standards. All phenolic compounds were expressed as mg/kg of fresh weight (grape berry).

### 3.6. Quantitative Analysis of Aroma Compounds

#### 3.6.1. Free-Form Aroma Compound Analysis by HS-SPME-GC-MS

The extraction and detection methods of aroma compounds in grape berries were performed according to our previous studies [38]. First, 50 grape berries were deseeded and then mixed with 1.0 g of PVPP and 0.5 g of d-gluconolactone. This mixture was ground to a powder in liquid nitrogen, macerated at 4 °C for 4 h and immediately centrifuged at 8000× *g* for 15 min to obtain a clear juice. A glass vial, containing 1 g of NaCl, 5 mL of clear juice and 10 µL of internal standard (1.0086 g/L 4-methyl-2-pentanol), was placed on a CTC CombiPAL autosampler (CTC Analytics, Zwingen, Switzerland). The sample vials were equilibrated at 40 °C for 30 min. Subsequently, DVB/CAR/PDMS 50/30 μm SPME fibres (Supelco, Bellefonte, PA, USA) were inserted into the headspace of the vials and stirred at 500 rpm for 30 min at 40 °C to extract volatile compounds. The SPME fibre underwent desorption in the injector for 8 min.

An Agilent 6890 gas chromatograph coupled with an Agilent 5975C mass spectrometer (Agilent Technologies) was used to perform GC-MS analysis. Volatile compounds were separated using an HP-INNOWAX capillary column (60 m × 0.25 mm × 0.25 μm, J&W Scientific, Folsom, CA, USA) with helium as the carrier gas at 1 mL/min flow rate. Injection was done in splitless mode with the injector temperature set at 250 °C. The column chamber was held at 50 °C for 1 min and then gradually heated at a rate of 3 °C/min until it reached 220 °C, at which point it was held for 5 min. The mass spectrometer utilizes electron ionization (EI) at 70 eV to ionize molecules of volatile compounds. The temperatures for the ion source and quadrupole were maintained at 250 °C and 150 °C, respectively. Full scan mode (*m*/*z* 30–350) was set for the mass detector.

#### 3.6.2. Bound-Form Volatile Compound Analysis by SPE-HS-SPME-GC-MS

Glycosylated aroma compounds were extracted from grapes via solid-phase extraction (SPE). Initially, the PEP–SEP column (150 mg/6 mL; Bonner Aguilar Technology Co., Ltd., Beijing, China) was subjected to pretreatment with 10 mL of methanol and 10 mL of water, which was followed by the addition of 2 mL of clear juice. Afterward, the cartridge was washed with 2 mL of water and 5 mL of dichloromethane to remove polar compounds and free aroma compounds. The bound aroma compounds were obtained through elution with 20 mL of methanol. The resulting methanol extract was then dried using a reduced-pressure evaporator (RayKol, Xiamen, China) until it was fully evaporated and was finally redissolved in 10 mL of citrate-phosphate buffer solution (0.2 M, pH = 5.0). Afterwards, 100 μL of AR2000 (fast enzyme, 100 g/L) was added to the buffer, and the mixture was subsequently incubated at 40 °C for 16 h for enzymatic hydrolysis. The sample preparation and HS-SPME-GC-MS analytic conditions were consistent with those used for the free aroma compounds.

#### 3.6.3. Identification and Quantification of Aroma Compounds

Aroma compounds were identified by comparing their retention indices (RIs) and mass spectra with those of reference standards and compounds from the NIST 11 MS database. Quantification of aroma compounds relied on calibration curves from reference standards, while compounds lacking standards were semi-quantified using calibration curves of structurally similar standards. Calibration curves were generated by conducting 10 serial dilutions in applicable synthetic matrix solutions. The synthetic matrix solution comprised 7 g/L tartaric acid and 200 g/L glucose and was pH-adjusted to 3.3 with NaOH.

### 3.7. Quantitative Analysis of Carotenoids

Carotenoids in grape berries were extracted according to the methods used in previous studies [39,40]. Frozen grape-berry powder (0.100 ± 0.002 g) was weighed and mixed with 1.0 mL Milli-Q water and 10 μL internal standard (20 µg/mL 8′-apo-*β*-carotenal). Subsequently, 1 mL diethyl ether/hexane (1:1) solution was added and the samples were subjected to shaking in the dark for 30 min and centrifugation at 12,000 *g* for 2 min. The extraction residue was collected twice, and the upper layer solution was collected and dried under nitrogen. The dried samples were dissolved in 200 μL methanol containing 0.1% (*w*/*v*) BHT, filtered through a 0.22 μm membrane filter, and then prepared for analysis.

Carotenoids in grapes were analysed using ultra-high performance liquid chromatography-triple quadrupole mass spectrometry (UHPLC-QqQ-MS/MS, Agilent 1290 Series UHPLC, Agilent 7670B QqQ-MS). The chromatographic method referred to the method provided by Agilent’s official website (https://www.agilent.com.cn/cs/library/applications/an-fat-soluble-carotenoid-infinityII-6470a-poroshell-5994-5064zh-cn-agilent.pdf, accessed on 20 July 2022) with some modifications. UHPLC separation was carried out on an Agilent ZORBAX RRHD Eclipse Plus 95Å PAH (1.8 μm, 100 mm × 2.1 mm, Agilent Technologies) column, with mobile phases (A) water containing 0.1% FA and (B) methanol containing 0.1% FA. Quantitative and qualitative analysis were performed using *β*-carotene, xanthophyll, zeaxanthin, antheraxanthin, violaxanthin, and neoxanthin standards. The specific chromatographic and mass spectrometric parameters were described in a previous article [40].

### 3.8. Statistical Analysis

Statistical analyses were conducted using version 3.4.2 of the R software package developed by the R Core Team (http://www.r-project.org/). A one-way ANOVA was applied to the physicochemical parameters and the aroma compound data for the grapes using Duncan’s multiple range test and a significance level of 5% (*p* ≤ 0.05).

## 4. Conclusions

This study indicates that cluster-zone leaf removal is an effective method for reducing acidity and significantly influences the composition of phenolic and volatile compounds in berries. The removal of cluster-zone leaves at the hard green berry stage, before and after veraison, generally increased the content of anthocyanins and flavonols, thereby improving the quality of the colour of the berries. The effect of cluster-zone leaf removal on the aroma of Cabernet Sauvignon grapes is twofold. The collective evidence from two years of experimentation suggests that early leaf removal increases the content of bound terpenes while decreasing norisoprenoid levels. This may be related to the reduction in neoxanthin content, the precursor of β-damascenone, which has a significant impact on the fruity aroma of the grape berry. Additionally, early leaf removal may also promote the transformation of C6/C9 aldehydes into alcohols, as well as a decrease in free-form terpenes. However, these findings were only observed in a single year and require further validation. As for Marselan grapes, leaf removal increases the levels of C6/C9 aldehydes but has limited effects on terpenes and norisoprenoids. These findings indicate that leaf removal after veraison can enhance the colour of Cabernet Sauvignon grapes without adversely affecting the quality of their aroma. In the case of Marselan grapes, leaf removal at the hard green berry stage can result in the highest concentration of anthocyanins and flavonols without affecting the aroma of the berries. However, it should be noted that our study on Marselan grapes was conducted only over one year, and thus the effects of leaf removal on Marselan grapes still require further examination.

## Figures and Tables

**Table 1:** Physicochemical parameters of grapes at harvest from vines at different stages of leaf-removal treatment from 2021 to 2022.

Vintage and Variety	Treatment	Weight of 100 Grapes (g)	Total Soluble Solids (°Brix)	pH Value	Titratable Acidity (g/L)
2021 Cabernet Sauvignon	LR1	128.40 ± 4.04	20.43 ± 0.5	3.37 ± 0.02 ab	7.26 ± 0.19 ab
	LR2	134.60 ± 12.15	19.97 ± 0.12	3.4 ± 0.03 a	6.82 ± 0.11 b
	LR3	139.93 ± 5.99	20.03 ± 0.45	3.39 ± 0.01 a	7.13 ± 0.34 ab
	None	133.60 ± 7.92	20.4 ± 0.44	3.32 ± 0.03 b	7.51 ± 0.14 a
	*p* value	ns	ns	0.0147	0.0252
2022 Cabernet Sauvignon	LR1	118.29 ± 2.76	20.3 ± 1.31	3.23 ± 0.02	4.38 ± 0.05 b
	LR2	126.09 ± 2.31	21.23 ± 0.42	3.26 ± 0.01	4.27 ± 0.08 b
	LR3	119.88 ± 3.39	20.97 ± 0.78	3.24 ± 0.01	4.29 ± 0.01 b
	None	126.13 ± 6.37	21.43 ± 0.75	3.21 ± 0.03	4.53 ± 0.06 a
	*p* value	ns	ns	ns	0.0018
2022 Marselan	LR1	112.70 ± 3.62	21.4 ± 0.36	3.37 ± 0.03	6.75 ± 0.11 b
	LR2	113.00 ± 1.22	21.63 ± 0.4	3.34 ± 0.03	6.94 ± 0.09 ab
	LR3	105.33 ± 4.4	21.07 ± 0.47	3.34 ± 0.03	6.82 ± 0.11 b
	None	106.93 ± 8.77	21.53 ± 0.06	3.35 ± 0.02	7.17 ± 0.36 a
	*p* value	ns	ns	ns	0.0124

Note: Lowercase letters indicate significant differences at *p* < 0.05 according to Tukey’s test, and “ns” indicates no statistical differences. The stages of leaf-removal treatment include the following: LR1 (cluster-zone leaf removal at grape-berry pea size), LR2 (cluster-zone leaf removal at the beginning of veraison), LR3 (cluster-zone leaf removal about 25 days after veraison) and None (no leaf removal).

**Table 2:** Composition of anthocyanins (mg/kg FW) in Cabernet Sauvignon and Marselan grapes at different stages of leaf-removal treatment from 2021 to 2022.

Compounds	Vintage and Variety	LR1	LR2	LR3	CK
Monomeric anthocyanins					
Cyanidin-3-*O*-glucoside	2021 Cabernet Sauvignon	223.05 ± 19.4 a	181.29 ± 10.79 b	223.4 ± 15.58 a	219.02 ± 4.21 a
	2022 Cabernet Sauvignon	214.15 ± 3.83 b	273.57 ± 8.91 a	216.1 ± 16.04 b	186.79 ± 4.7 c
	2022 Marselan	187.29 ± 8 a	152.68 ± 2.52 b	187.03 ± 7.31 a	152.55 ± 6.58 b
Peonidin-3-*O*-glucoside	2021 Cabernet Sauvignon	624.8 ± 21.57 a	558.62 ± 14.1 b	579.79 ± 26.91 b	567.67 ± 4.17 b
	2022 Cabernet Sauvignon	518.32 ± 5.72 b	572.32 ± 7.23 a	497.24 ± 17.02 c	504.31 ± 4.66 bc
	2022 Marselan	408.65 ± 8.92 a	377.38 ± 2.46 b	402.11 ± 3.63 a	331.67 ± 9.6 c
Delphinidin-3-*O*-glucoside	2021 Cabernet Sauvignon	651.97 ± 59.17	608.53 ± 43.32	649.67 ± 58.29	657.27 ± 25.22
	2022 Cabernet Sauvignon	636.64 ± 31.18 b	788.8 ± 32.27 a	590.21 ± 38.14 b	600.22 ± 21.39 b
	2022 Marselan	669.39 ± 64.53 a	523.95 ± 21.05 bc	572.28 ± 36.71 b	456.52 ± 31.79 c
Petunidin-3-*O*-glucoside	2021 Cabernet Sauvignon	343.8 ± 26.4	334.12 ± 16.98	353.27 ± 32.2	340.53 ± 7.94
	2022 Cabernet Sauvignon	357.85 ± 10.45 b	424.37 ± 13.65 a	322.91 ± 16.44 c	331.18 ± 9.1 c
	2022 Marselan	560.93 ± 41.04 a	470.3 ± 11.56 bc	503.81 ± 32.49 b	427.25 ± 13.24 c
Malvidin-3-*O*-glucoside	2021 Cabernet Sauvignon	1822.93 ± 64.3 a	1919.16 ± 53.43 a	1803.66 ± 84.17 a	1673.74 ± 11.37 b
	2022 Cabernet Sauvignon	1937.1 ± 37.06 b	2035.78 ± 39.18 a	1723.4 ± 36.79 d	1844.14 ± 25.95 c
	2022 Marselan	2767.63 ± 58.28 a	2774.62 ± 25.77 a	2767.31 ± 68.72 a	2634.22 ± 55.26 b
Acylated anthocyanins					
Cyanidin-3-*O*-(6-*O*-acetyl) glucoside	2021 Cabernet Sauvignon	39.16 ± 4.52 a	27.77 ± 1.54 b	39.87 ± 4.37 a	38.67 ± 1.16 a
	2022 Cabernet Sauvignon	33.45 ± 1.61 b	43.88 ± 0.71 a	31.29 ± 3.51 bc	28.13 ± 1.65 c
	2022 Marselan	21.24 ± 1.81 a	14.99 ± 0.49 b	20.77 ± 2.17 a	14.33 ± 1.26 b
Peonidin-3-*O*-(6-*O*-acetyl) glucoside	2021 Cabernet Sauvignon	238.9 ± 8.33 a	221.38 ± 4.73 b	239.26 ± 12.19 a	220.04 ± 1.6 b
	2022 Cabernet Sauvignon	215.5 ± 2.98 b	233.56 ± 2.54 a	199.54 ± 7.32 c	209.89 ± 3.58 b
	2022 Marselan	167.69 ± 5.03 a	157.67 ± 0.52 b	159.18 ± 6.45 b	128.69 ± 1.9 c
Delphinidin-3-*O*-(6-*O*-acetyl) glucoside	2021 Cabernet Sauvignon	142.43 ± 13.39	136.38 ± 7.4	146.51 ± 14.88	148.5 ± 2.63
	2022 Cabernet Sauvignon	141.68 ± 6.01 b	174.29 ± 7.49 a	128.87 ± 9.13 b	130.58 ± 3.03 b
	2022 Marselan	115.2 ± 12.15 a	92.67 ± 4.73 b	99.13 ± 9.81 ab	72.28 ± 6.72 c
Petunidin-3-*O*-(6-*O*-acetyl) glucoside	2021 Cabernet Sauvignon	196.67 ± 18.36	190.03 ± 9.96	214.61 ± 22.59	197.62 ± 4.16
	2022 Cabernet Sauvignon	213.62 ± 8.67 b	245.08 ± 7.28 a	177.87 ± 14.37 c	186.02 ± 8.41 c
	2022 Marselan	232.46 ± 15.14 a	195.91 ± 8.16 bc	215.55 ± 18.98 ab	173.26 ± 7.34 c
Malvidin-3-*O*-(6-*O*-acetyl) glucoside	2021 Cabernet Sauvignon	1397.61 ± 57 a	1426.52 ± 38.75 a	1407.32 ± 80.01 a	1231.66 ± 5.99 b
	2022 Cabernet Sauvignon	1602.35 ± 47.26 a	1628.64 ± 32.29 a	1390.87 ± 36.59 c	1472.92 ± 30.21 b
	2022 Marselan	1789.73 ± 42.95 b	1875.68 ± 37.02 a	1774.26 ± 51.32 bc	1702.95 ± 22.29 c
Coumaroylated anthocyanins					
Cyanidin-3-*O*-(6-*O*-*p*-coumaryl) glucoside	2021 Cabernet Sauvignon	8.85 ± 1.15	8.87 ± 0.91	9.51 ± 1.14	9.09 ± 0.59
	2022 Cabernet Sauvignon	12.56 ± 0.29 b	15.56 ± 0.63 a	10.65 ± 1.48 c	8.55 ± 0.59 d
	2022 Marselan	30.74 ± 2.59 a	20.1 ± 0.9 c	27.2 ± 1.6 b	20.54 ± 0.35 c
Peonidin-3-*O*-(6-*O*-*p*-coumaryl) glucoside	2021 Cabernet Sauvignon	139.2 ± 6.84 a	149.23 ± 7.46 a	143.31 ± 7.58 a	121.44 ± 1.1 b
	2022 Cabernet Sauvignon	146.4 ± 3.2 a	152.98 ± 4.59 a	126.22 ± 7.02 b	123.6 ± 3.55 b
	2022 -	233.78 ± 5.14 a	219.18 ± 1.68 b	224.19 ± 7.14 b	186.85 ± 0.67 c
Delphinidin-3-*O*-(6-*O*-*p*-coumaryl) glucoside	2021 Cabernet Sauvignon	0.4 ± 0.24 c	2.4 ± 0.19 a	1.04 ± 0.19 b	1.39 ± 0.21 b
	2022 Cabernet Sauvignon	0.34 ± 0.95 c	3.14 ± 0.78 a	1.95 ± 0.96 ab	1.17 ± 0.34 bc
	2022 Marselan	27.29 ± 3.84 a	20.3 ± 1.12 b	21.6 ± 1.32 b	16.13 ± 1.12 c
Petunidin-3-*O*-(6-*O*-*p*-coumaryl) glucoside	2021 Cabernet Sauvignon	25.39 ± 2.39 bc	33.4 ± 2.8 a	29.45 ± 3.04 ab	24.04 ± 0.87 c
	2022 Cabernet Sauvignon	34.52 ± 1.64 b	38.8 ± 1.86 a	29.53 ± 2.49 c	27.07 ± 0.99 c
	2022 Marselan	160.39 ± 16.73 a	142.61 ± 4.05 ab	142.03 ± 7.41 ab	125.78 ± 3.21 b
Malvidin-3-*O*-(6-*O*-*p*-coumaryl) glucoside	2021 Cabernet Sauvignon	458.99 ± 22.13 b	524.69 ± 31.73 a	491.13 ± 26.8 ab	390.34 ± 10.33 c
	2022 Cabernet Sauvignon	537.49 ± 13.06 a	525.23 ± 18.11 a	444.06 ± 25.78 b	446.76 ± 10.79 b
	2022 Marselan	1373.12 ± 64.74 b	1463.14 ± 33.96 a	1350.81 ± 31.7 b	1329.69 ± 28.43 b
Total anthocyanins	2021 Cabernet Sauvignon	6314.18 ± 321.38	6322.37 ± 231.46	6331.81 ± 386.6	5841.04 ± 64.99
	2022 Cabernet Sauvignon	6601.96 ± 159.33 b	7156 ± 163.02 a	5890.69 ± 224.22 c	6101.32 ± 120.66 c
	2022 Marselan	8745.53 ± 330.49 a	8501.18 ± 112.14 a	8467.28 ± 280.59 a	7772.72 ± 139.37 b
Acetylation rate (%)	2021 Cabernet Sauvignon	31.91 ± 0.11 ab	31.67 ± 0.21 b	32.13 ± 0.18 a	31.44 ± 0.12 b
	2022 Cabernet Sauvignon	33.42 ± 0.27 a	32.5 ± 0.07 c	32.71 ± 0.21 bc	33.23 ± 0.12 ab
	2022 Marselan	26.6 ± 0.15 b	26.91 ± 0.16 b	26.79 ± 0.12 b	27.49 ± 0.15 a
Coumaroylation rate (%)	2021 Cabernet Sauvignon	10.02 ± 0.01 c	11.36 ± 0.24 a	10.82 ± 0.17 b	9.35 ± 0.09 d
	2022 Cabernet Sauvignon	11.08 ± 0.03 a	10.28 ± 0.14 b	10.35 ± 0.24 b	9.95 ± 0.14 b
	2022 Marselan	20.87 ± 0.25 b	21.6 ± 0.33 ab	21.27 ± 0.44 b	21.94 ± 0.21 a
Methylation rate (%)	2021 Cabernet Sauvignon	83.14 ± 0.57 b	84.74 ± 0.35 a	83.98 ± 0.88 b	81.62 ± 0.27 c
	2022 Cabernet Sauvignon	84.27 ± 0.27 ab	81.85 ± 0.27 c	82.79 ± 0.92 b	84.34 ± 0.17 a
	2022 Marselan	88 ± 0.52 b	90.58 ± 0.39 a	89.82 ± 0.82 b	90.3 ± 0.23 a

Note: Mean ± SD are presented (*n* = 3); Lowercase letters indicate significant differences at *p* < 0.05 according to Tukey’s test.

**Table 3:** Composition of flavonols (mg/kg FW) in Cabernet Sauvignon and Marselan grapes at different stages of leaf-removal treatment from 2021 to 2022.

Compounds	Vintage and Variety	LR1	LR2	LR3	CK
kaempferol-3-*O*-glucoside	2021 Cabernet Sauvignon	3.05 ± 0.09 b	3.97 ± 0.36 a	3.77 ± 0.48 a	2.21 ± 0.07 c
	2022 Cabernet Sauvignon	8.38 ± 0.4 b	8.38 ± 0.32 b	9.18 ± 0.44 a	5.86 ± 0.46 c
	2022 Marselan	4.74 ± 0.39 a	3.96 ± 0.34 bc	4.48 ± 0.45 ab	3.74 ± 0.3 c
kaempferol-3-*O*-galactoside	2021 Cabernet Sauvignon	0.78 ± 0.03 b	0.92 ± 0.05 a	0.92 ± 0.07 a	0.51 ± 0.01 c
	2022 Cabernet Sauvignon	2.28 ± 0.15 b	2.29 ± 0.12 b	2.57 ± 0.18 a	1.46 ± 0.1 c
	2022 Marselan	1.25 ± 0.1 a	0.96 ± 0.01 bc	1.13 ± 0.16 ab	0.91 ± 0.09 c
kaempferol-3-*O*-glucuronide	2021 Cabernet Sauvignon	0.48 ± 0.01 b	0.54 ± 0.05 a	0.43 ± 0.03 b	0.36 ± 0.02 c
	2022 Cabernet Sauvignon	1.06 ± 0.05 a	1.13 ± 0.05 a	1.05 ± 0.08 a	0.73 ± 0.06 b
	2022 Marselan	0.47 ± 0.03 a	0.41 ± 0.01 b	0.41 ± 0.05 ab	0.38 ± 0.02 b
quercetin-3-*O*-glucoside	2021 Cabernet Sauvignon	20.48 ± 0.36 b	24.65 ± 1.48 a	23.22 ± 1.21 a	17.29 ± 0.38 c
	2022 Cabernet Sauvignon	32.57 ± 0.96 a	32.19 ± 1.02 a	28.89 ± 1.02 b	24.57 ± 1.34 c
	2022 Marselan	28.54 ± 0.86 a	24.86 ± 0.72 b	25.41 ± 1.45 b	24.45 ± 0.91 b
quercetin-3-*O*-galactoside	2021 Cabernet Sauvignon	6.99 ± 0.36 b	9.23 ± 0.84 a	8.28 ± 0.56 a	5.39 ± 0.13 c
	2022 Cabernet Sauvignon	16.05 ± 0.87 a	15.36 ± 0.8 ab	13.85 ± 0.76 b	10.43 ± 0.96 c
	2022 Marselan	6.95 ± 0.62 a	5.26 ± 0.51 b	5.6 ± 0.98 b	4.94 ± 0.47 b
quercetin-3-*O*-glucuronide	2021 Cabernet Sauvignon	20.17 ± 0.53	22.06 ± 1.69	19.4 ± 1.68	19.98 ± 1.47
	2022 Cabernet Sauvignon	34.9 ± 0.74 a	34.89 ± 1.2 a	28.42 ± 1.75 b	27.39 ± 1.73 b
	2022 Marselan	28.55 ± 0.64 a	23.33 ± 0.24 c	22.21 ± 2.32 c	26.09 ± 0.51 b
quercetin-3-*O*-rhamnoside	2021 Cabernet Sauvignon	nd	nd	nd	nd
	2022 Cabernet Sauvignon	4.7 ± 0.37 b	5.63 ± 0.25 a	3.21 ± 0.54 c	1.67 ± 0.39 d
	2022 Marselan	6.33 ± 0.59 a	6.74 ± 0.34 a	4.04 ± 1.07 b	4.19 ± 0.22 b
quercetin-3-*O*-rutinoside	2021 Cabernet Sauvignon	0.9 ± 0.01 b	0.89 ± 0.09 b	0.89 ± 0.09 b	1.17 ± 0.06 a
	2022 Cabernet Sauvignon	2.71 ± 0.12 a	2.37 ± 0.14 b	2.16 ± 0.2 bc	1.96 ± 0.24 c
	2022 Marselan	3.21 ± 0.15 a	2.09 ± 0.04 b	2.15 ± 0.23 b	2.36 ± 0.04 b
Isorhamnetin-3-*O*-glucoside	2021 Cabernet Sauvignon	4.79 ± 0.1 c	6.41 ± 0.48 a	5.69 ± 0.37 b	4.16 ± 0.26 c
	2022 Cabernet Sauvignon	7.43 ± 0.23 a	7.32 ± 0.27 a	7.08 ± 0.28 a	5.37 ± 0.38 b
	2022 Marselan	6.76 ± 0.2 a	5.89 ± 0.26 bc	5.4 ± 0.48 c	6.14 ± 0.25 b
Isorhamnetin-3-*O*-glucuronide	2021 Cabernet Sauvignon	0.95 ± 0.05 b	1.17 ± 0.11 a	0.97 ± 0.08 b	nd
	2022 Cabernet Sauvignon	1.51 ± 0.07 ab	1.54 ± 0.09 a	1.36 ± 0.07 b	0.93 ± 0.1 c
	2022 Marselan	2.91 ± 0.09 a	2.4 ± 0.3 ab	1.9 ± 0.21 b	2.71 ± 0.44 a
myricetin-3-*O*-glucoside	2021 Cabernet Sauvignon	43.68 ± 0.6 b	54.96 ± 5.22 a	52.62 ± 4.67 a	36.84 ± 2.55 b
	2022 Cabernet Sauvignon	64.47 ± 3 a	62.18 ± 3.83 ab	57.47 ± 3.77 b	46.95 ± 2.62 c
	2022 Marselan	74.74 ± 5.29 a	56.31 ± 4.17 b	61.59 ± 3.43 b	70.87 ± 2.47 a
myricetin-3-*O*-galactoside	2021 Cabernet Sauvignon	1.4 ± 0.1 a	1.67 ± 0.2 a	1.69 ± 0.19 a	0.98 ± 0.08 b
	2022 Cabernet Sauvignon	2.5 ± 0.12 a	2.34 ± 0.11 a	2 ± 0.03 b	1.58 ± 0.24 c
	2022 Marselan	1.84 ± 0.1 a	1.14 ± 0.14 c	1.31 ± 0.12 c	1.59 ± 0.06 b
myricetin-3-*O*-glucuronide	2021 Cabernet Sauvignon	3.9 ± 0.04 b	4.53 ± 0.39 a	3.94 ± 0.33 b	3.31 ± 0.25 c
	2022 Cabernet Sauvignon	6.14 ± 0.25 a	6.04 ± 0.29 a	5.27 ± 0.42 b	4.46 ± 0.26 c
	2022 Marselan	5.93 ± 0.46 a	4.16 ± 0.21 b	4.36 ± 0.4 b	5.59 ± 0.17 a
laricitrin-3-*O*-glucoside	2021 Cabernet Sauvignon	3.18 ± 0.05 bc	4.18 ± 0.37 a	3.61 ± 0.21 b	2.81 ± 0.19 c
	2022 Cabernet Sauvignon	5.23 ± 0.15 a	4.57 ± 0.19 b	4.53 ± 0.23 b	3.83 ± 0.24 c
	2022 Marselan	7.48 ± 0.37 a	6.62 ± 0.31 b	6.27 ± 0.41 b	7.45 ± 0.36 a
syringetin-3-*O*-glucoside	2021 Cabernet Sauvignon	2.32 ± 0.01 b	2.96 ± 0.21 a	2.29 ± 0.16 b	2.22 ± 0.17 b
	2022 Cabernet Sauvignon	3.21 ± 0.1 a	2.66 ± 0.08 b	2.58 ± 0.11 bc	2.41 ± 0.14 c
	2022 Marselan	6.41 ± 0.33 ab	5.95 ± 0.28 bc	5.44 ± 0.4 c	7.07 ± 0.45 a
Total flavonols	2021 Cabernet Sauvignon	113.06 ± 1.73 b	138.14 ± 11.45 a	127.73 ± 10 ab	97.23 ± 5.41 c
	2022 Cabernet Sauvignon	193.14 ± 7.38 a	188.88 ± 8.17 a	169.61 ± 9.79 b	139.61 ± 9.04 c
	2022 Marselan	186.13 ± 4.59 a	150.08 ± 6.81 c	151.7 ± 11.47 c	168.47 ± 6.18 b
Proportion of kaempferol flavonols (%)	2021 Cabernet Sauvignon	3.81 ± 0.04 a	3.93 ± 0.02 a	4 ± 0.16 a	3.19 ± 0.19 b
	2022 Cabernet Sauvignon	6.07 ± 0.06 b	6.24 ± 0.1 b	7.55 ± 0.04 a	5.77 ± 0.06 c
	2022 Marselan	3.47 ± 0.28 ab	3.55 ± 0.09 a	3.96 ± 0.17 a	2.98 ± 0.11 b
Proportion of quercetin flavonols (%)	2021 Cabernet Sauvignon	42.94 ± 0.33 b	41.17 ± 0.39 c	40.57 ± 0.4 c	45.09 ± 0.52 a
	2022 Cabernet Sauvignon	47.09 ± 0.26 a	47.89 ± 0.53 a	45.12 ± 0.09 b	47.28 ± 0.3 a
	2022 Marselan	39.56 ± 1.64 a	41.52 ± 0.82 a	39.11 ± 0.91 a	36.83 ± 0.15 b
Proportion of Isorhamnetin flavonols (%)	2021 Cabernet Sauvignon	5.08 ± 0.06 b	5.48 ± 0.04 a	5.22 ± 0.05 b	4.28 ± 0.07 c
	2022 Cabernet Sauvignon	4.63 ± 0.03 b	4.69 ± 0.06 b	4.98 ± 0.09 a	4.51 ± 0.04 b
	2022 Marselan	5.2 ± 0.16 a	5.53 ± 0.27 a	4.81 ± 0.09 b	5.25 ± 0.17 a
Proportion of myricetin flavonols (%)	2021 Cabernet Sauvignon	43.31 ± 0.41 bc	44.25 ± 0.43 ab	45.58 ± 0.41 a	42.28 ± 0.58 c
	2022 Cabernet Sauvignon	37.85 ± 0.26	37.34 ± 0.67	38.15 ± 0.21	37.97 ± 0.41
	2022 Marselan	44.3 ± 1.89 ab	41.02 ± 1.19 b	44.39 ± 1.11 ab	46.33 ± 0.31 a
Proportion of laricitrin flavonols (%)	2021 Cabernet Sauvignon	2.82 ± 0.02 b	3.02 ± 0.03 a	2.83 ± 0.05 b	2.89 ± 0.05 b
	2022 Cabernet Sauvignon	2.71 ± 0.04 a	2.42 ± 0.01 b	2.67 ± 0.03 a	2.75 ± 0.04 a
	2022 Marselan	4.02 ± 0.09 b	4.41 ± 0.05 a	4.13 ± 0.04 b	4.42 ± 0.13 a
Proportion of syringetin flavonols (%)	2021 Cabernet Sauvignon	2.05 ± 0.03 b	2.15 ± 0.03 b	1.8 ± 0.03 c	2.28 ± 0.04 a
	2022 Cabernet Sauvignon	1.66 ± 0.02 a	1.41 ± 0.03 c	1.52 ± 0.02 b	1.73 ± 0.04 a
	2022 Marselan	3.44 ± 0.11 b	3.97 ± 0.1 a	3.59 ± 0.07 b	4.19 ± 0.15 a

Note: Mean ± SD are presented (*n* = 3); “nd” indicate “not detected”. Lowercase letters indicate significant differences at *p* < 0.05 according to Tukey’s test.

**Table 5:** Composition of terpenes (μg/kg) in Cabernet Sauvignon and Marselan grapes at different stages of leaf-removal treatment from 2021 to 2022.

Compounds	Vintage and Variety	LR1	LR2	LR3	CK
*p*-Cymene	2021 Cabernet Sauvignon	0.47 ± 0.17	0.35 ± 0.13	0.54 ± 0.21	0.38 ± 0.11
	2022 Cabernet Sauvignon	0.14 ± 0.02 b	0.3 ± 0.09 a	0.3 ± 0.11 a	0.37 ± 0.06 a
	2022 Marselan	1.87 ± 0.86	1.78 ± 0.96	1.67 ± 0.91	0.9 ± 0.27
*p*-Cymenene	2021 Cabernet Sauvignon	0.12 ± 0.01	0.12 ± 0.01	0.14 ± 0.02	0.13 ± 0.01
	2022 Cabernet Sauvignon	0.1 ± 0.01 b	0.13 ± 0.01 a	0.13 ± 0.01 a	0.14 ± 0.02 a
	2022 Marselan	0.2 ± 0.02	0.22 ± 0.05	0.22 ± 0.02	0.17 ± 0.02
Linalol	2021 Cabernet Sauvignon	0.11 ± 0.01 ab	0.1 ± 0.01 b	0.09 ± 0 b	0.14 ± 0.03 a
	2022 Cabernet Sauvignon	nd	nd	nd	nd
	2022 Marselan	0.12 ± 0.02	0.16 ± 0.05	0.17 ± 0.04	0.17 ± 0.02
Levomenthol	2021 Cabernet Sauvignon	0.72 ± 0.08	0.48 ± 0.07	0.61 ± 0.11	0.63 ± 0.18
	2022 Cabernet Sauvignon	0.47 ± 0.06 b	0.54 ± 0.11 ab	0.47 ± 0.03 b	0.71 ± 0.18 a
	2022 Marselan	0.64 ± 0.09 a	0.44 ± 0.02 b	0.42 ± 0.04 b	0.41 ± 0.1 b
*α*-Terpineol	2021 Cabernet Sauvignon	0.06 ± 0.01 b	0.05 ± 0.01 b	0.07 ± 0.01 b	0.09 ± 0.01 a
	2022 Cabernet Sauvignon	nd	nd	nd	nd
	2022 Marselan	0.08 ± 0.01	0.11 ± 0.02	0.11 ± 0.01	0.1 ± 0.03
*γ*-Terpineol	2021 Cabernet Sauvignon	0.04 ± 0.01 b	0.03 ± 0.01 b	0.04 ± 0.01 b	0.06 ± 0.01 a
	2022 Cabernet Sauvignon	nd	nd	nd	nd
	2022 Marselan	0.07 ± 0.02	0.09 ± 0.02	0.09 ± 0.01	0.07 ± 0.01
Levomenthol (Bound)	2021 Cabernet Sauvignon	2.37 ± 0.43	2.21 ± 0.23	2.03 ± 0.19	1.99 ± 0.37
	2022 Cabernet Sauvignon	2.92 ± 0.08 a	1.68 ± 0.03 b	2.64 ± 0.21 ab	1.68 ± 0.15 b
	2022 Marselan	1.52 ± 0.05	2.01 ± 0.62	1.45 ± 0.09	2.02 ± 0.73
*α*-Terpineol (Bound)	2021 Cabernet Sauvignon	0.32 ± 0.04	0.26 ± 0.05	0.25 ± 0.03	0.28 ± 0.08
	2022 Cabernet Sauvignon	0.27 ± 0.08 a	0.1 ± 0.04 b	0.2 ± 0.06 ab	0.13 ± 0.07 ab
	2022 Marselan	0.37 ± 0.09	0.39 ± 0.03	0.48 ± 0.17	0.31 ± 0.07
*β*-Citronellol (Bound)	2021 Cabernet Sauvignon	0.17 ± 0.03	0.15 ± 0.01	0.14 ± 0.01	0.17 ± 0.04
	2022 Cabernet Sauvignon	nd	nd	nd	nd
	2022 Marselan	0.22 ± 0.05	0.37 ± 0.11	0.29 ± 0.08	0.34 ± 0.07
Total Terpenes (Free)	2021 Cabernet Sauvignon	1.52 ± 0.22	1.13 ± 0.17	1.49 ± 0.27	1.45 ± 0.11
	2022 Cabernet Sauvignon	0.71 ± 0.06 b	0.98 ± 0.05 ab	0.9 ± 0.12 ab	1.23 ± 0.18 a
	2022 Marselan	2.98 ± 0.71	2.8 ± 0.87	2.68 ± 0.75	1.81 ± 0.36
Total Terpenes (Bound)	2021 Cabernet Sauvignon	3.24 ± 0.27 a	2.62 ± 0.16 ab	2.43 ± 0.18 b	2.36 ± 0.32 b
	2022 Cabernet Sauvignon	3.2 ± 0.78 a	1.78 ± 0.03 b	2.85 ± 0.21 ab	1.81 ± 0.18 b
	2022 Marselan	2.12 ± 0.09	2.76 ± 0.62	2.23 ± 0.15	2.67 ± 0.67
Total Terpenes	2021 Cabernet Sauvignon	4.76 ± 0.41	3.75 ± 0.33	3.92 ± 0.36	3.81 ± 0.4
	2022 Cabernet Sauvignon	3.91 ± 0.73	2.76 ± 0.05	3.75 ± 0.31	3.03 ± 0.34
	2022 Marselan	5.1 ± 0.67	5.56 ± 0.35	4.91 ± 0.66	4.47 ± 0.4

Note: Mean ± SD are presented (*n* = 3); “nd” indicate “not detected”. Lowercase letters indicate significant differences at *p* < 0.05 according to Tukey’s test.

**Table 6:** Composition of norisoprenoids (μg/kg) and carotenoids (mg/kg) in Cabernet Sauvignon and Marselan grapes at different stages of leaf-removal treatment from 2021 to 2022.

Compounds	Vintage and Variety	LR1	LR2	LR3	CK
Norisoprenoids					
6-methyl-5-Hepten-2-one	2021 Cabernet Sauvignon	0.31 ± 0.07 a	0.21 ± 0.04 b	0.3 ± 0.02 a	0.27 ± 0.02 ab
	2022 Cabernet Sauvignon	0.17 ± 0.02 b	0.2 ± 0.01 b	0.26 ± 0.09 ab	0.33 ± 0.06 a
	2022 Marselan	0.32 ± 0.07	0.25 ± 0.05	0.29 ± 0.08	0.23 ± 0.02
Vitispirane	2021 Cabernet Sauvignon	nd	nd	nd	0.04 ± 0.02
	2022 Cabernet Sauvignon	nd	0.04 ± 0.02 b	0.04 ± 0.02 b	0.15 ± 0.03 a
	2022 Marselan	0.62 ± 0.29	0.93 ± 0.27	0.81 ± 0.04	0.93 ± 0.36
*β*-Ionone	2021 Cabernet Sauvignon	0.09 ± 0.02	0.1 ± 0.02	0.1 ± 0.01	0.11 ± 0
	2022 Cabernet Sauvignon	0.07 ± 0.01 b	0.07 ± 0.01 b	0.07 ± 0 b	0.12 ± 0.03 a
	2022 Marselan	0.08 ± 0.01	0.07 ± 0.01	0.07 ± 0.01	0.06 ± 0.01
Theaspirane	2021 Cabernet Sauvignon	nd	nd	nd	nd
	2022 Cabernet Sauvignon	nd	nd	nd	nd
	2022 Marselan	0.36 ± 0.08	0.27 ± 0.07	0.32 ± 0.05	0.29 ± 0.06
*β*-Damascenone	2021 Cabernet Sauvignon	1.78 ± 0.59 c	3.15 ± 0.93 c	6.94 ± 1.26 b	11.3 ± 2.08 a
	2022 Cabernet Sauvignon	1.67 ± 1.09 c	4.85 ± 2.15 bc	6.28 ± 2.11 b	14.03 ± 1.39 a
	2022 Marselan	19.02 ± 3.97	23.57 ± 0.87	25.62 ± 4.6	21.89 ± 5.63
*cis*-Geranyl acetone	2021 Cabernet Sauvignon	0.34 ± 0.08	0.27 ± 0.06	0.33 ± 0.08	0.27 ± 0.03
	2022 Cabernet Sauvignon	0.13 ± 0.03 b	0.18 ± 0.04 b	0.23 ± 0.09 ab	0.46 ± 0.25 a
	2022 Marselan	0.48 ± 0.1	0.39 ± 0.06	0.33 ± 0.17	0.32 ± 0.12
*β*-Damascenone (Bound)	2021 Cabernet Sauvignon	nd	nd	nd	nd
	2022 Cabernet Sauvignon	0.32 ± 0.15 a	0.11 ± 0.02 b	0.32 ± 0.04 a	0.22 ± 0.05 ab
	2022 Marselan	0.21 ± 0.04 b	0.45 ± 0.13 a	0.42 ± 0.04 a	0.53 ± 0.12 a
Carotenoids					
*β*-catotene	2021 Cabernet Sauvignon	3.28 ± 0.09 b	3.18 ± 0.33 b	3.78 ± 0.23 a	3.5 ± 0.21 ab
	2022 Cabernet Sauvignon	4.88 ± 0.58	4.89 ± 0.62	3.75 ± 0.42	4.35 ± 0.81
	2022 Marselan	6.46 ± 0.43 ab	5.99 ± 0.5 b	5.88 ± 0.26 b	7.31 ± 0.21 a
Xanthophyll	2021 Cabernet Sauvignon	24.55 ± 6.35	24.27 ± 1.47	29.7 ± 0.18	27.54 ± 3.26
	2022 Cabernet Sauvignon	33.58 ± 7.2 a	36.04 ± 3.37 a	25.65 ± 0.97 b	25.75 ± 2.1 b
	2022 Marselan	31.83 ± 5.03	31.76 ± 4.16	32.58 ± 4.01	39.23 ± 2.67
Zeaxanthin	2021 Cabernet Sauvignon	2.84 ± 0.51 a	1.79 ± 0.14 c	2.64 ± 0.31 ab	2.11 ± 0.03 bc
	2022 Cabernet Sauvignon	5.17 ± 0.57	5.27 ± 0.73	4 ± 0.75	4.85 ± 0.84
	2022 Marselan	3.79 ± 0.43 ab	3.27 ± 0.42 b	3.63 ± 0.38 ab	4.31 ± 0.28 a
Antheraxanthin	2021 Cabernet Sauvignon	0.5 ± 0.13	0.47 ± 0.16	0.62 ± 0.04	0.56 ± 0.17
	2022 Cabernet Sauvignon	1.15 ± 0.2	1.09 ± 0.33	0.82 ± 0.08	1.17 ± 0.2
	2022 Marselan	1.13 ± 0.15	1.02 ± 0.09	1.09 ± 0.23	1.16 ± 0.08
Violaxanthin	2021 Cabernet Sauvignon	1.07 ± 0.12 b	1.22 ± 0.35 ab	1.55 ± 0.43 ab	1.87 ± 0.38 a
	2022 Cabernet Sauvignon	1.81 ± 0.32	1.88 ± 0.79	1.91 ± 0.6	2.29 ± 1.15
	2022 Marselan	3.99 ± 1.65	3.23 ± 0.86	2.68 ± 0.08	3.17 ± 0.44
Neoxanthin	2021 Cabernet Sauvignon	1.07 ± 0.26 b	1.1 ± 0.1 b	1.44 ± 0.29 ab	1.9 ± 0.21 a
	2022 Cabernet Sauvignon	1.13 ± 0.17	1.38 ± 0.21	1.43 ± 0.32	1.57 ± 0.51
	2022 Marselan	2.82 ± 1.13	2.39 ± 0.98	2.15 ± 0.07	3.03 ± 0.24
Total Norisoprenoids	2021 Cabernet Sauvignon	2.55 ± 0.4 c	3.77 ± 0.75 c	7.72 ± 0.97 b	12.02 ± 1.66 a
	2022 Cabernet Sauvignon	2.4 ± 0.96 b	5.48 ± 1.77 b	7.23 ± 1.78 b	15.33 ± 1.38 a
	2022 Marselan	21.1 ± 3.58	25.93 ± 0.56	27.85 ± 3.71	24.25 ± 4.78
Total carotenoids	2021 Cabernet Sauvignon	33.31 ± 6.8	32.03 ± 1.62	39.72 ± 0.22	37.46 ± 3.88
	2022 Cabernet Sauvignon	47.72 ± 6.77 ab	50.55 ± 4.55 a	37.57 ± 2.54 b	39.97 ± 1.75 ab
	2022 Marselan	50 ± 6.17	47.66 ± 6.91	48.01 ± 4.78	58.21 ± 2.87
V/(V + A + Z) (%) ^a^	2021 Cabernet Sauvignon	24.63 ± 2.81	35.03 ± 7.57	32.13 ± 6.44	39.82 ± 8.16
	2022 Cabernet Sauvignon	22.24 ± 2.5	22.22 ± 3.38	28.01 ± 2.58	27.43 ± 10.97
	2022 Marselan	43.75 ± 9.57	42.53 ± 3.36	36.36 ± 2.96	36.57 ± 4.29

Note: Mean ± SD are presented (*n* = 3); “nd” indicates “not detected”. Lowercase letters indicate significant differences at *p* < 0.05 according to Tukey’s test. a: The equation represents the proportion of violaxanthin in the xanthophyll cycle. V: violaxanthin; A: antheraxanthin; Z: zeaxanthin.

## Data Availability

Data are contained within the article or Supplementary Materials.

## Supplementary Materials

The following supporting information can be downloaded at: https://www.mdpi.com/article/10.3390/plants13111543/s1, Table S1: Weather conditions in the vineyard during different phenological stages from 2021 to 2022; Table S2: Composition of flavan-3-ols (mg/kg FW) in Cabernet Sauvignon and Marselan grapes at different stages of leaf-removal treatment from 2021 to 2022; Table S3: Composition of aromatic compounds (μg/kg) in Cabernet Sauvignon and Marselan grapes at different stages of leaf-removal treatment from 2021 to 2022.
